# Return to Sport After Multiligament Knee Injury: A Systematic Review of the Literature

**DOI:** 10.1007/s43465-024-01237-w

**Published:** 2024-08-19

**Authors:** Riccardo D’Ambrosi, Amit Meena, Nicola Ursino, Fabrizio Di Feo, Niccolò Fusari, Srinivas B. S. Kambhampati

**Affiliations:** 1 IRCCS Ospedale Galeazzi–Sant’Ambrogio, Milan, Italy; 2 Dipartimento Di Scienze Biomediche Per La Salute, Università Degli Studi Di Milano, Milan, Italy; 3 Shalby Hospital, Jaipur, India; 4 Università Di Pisa, Pisa, Italy; 5 Maternity and Gynaecology Center, Sri Dhaatri Orthopaedic, SKDGOC, Vijayawada, Andhra Pradesh India

**Keywords:** Multiligament knee injuries, ACL, PCL, MCL, LCL, Return to sport

## Abstract

**Purpose:**

The objective of this study was to conduct a comprehensive assessment of MLKI outcome studies in order to ascertain the overall rates of return to sport following MLKI.

**Methods:**

A systematic review was conducted based on the PRISMA guidelines. Quality assessment of the systematic review was performed using the MINORS Score. The following search terms were browsed in the title, abstract, and keyword fields: “multiligament knee” or “MLKI" AND “return to sport” or “sports activity” or "athletes" or "sports" or "sportsman". The resulting measures extracted from the studies were the rate of RTS, level of RTS, complications, revision surgery, Tegner, International Knee Documentation Committee (IKDC) Lysholm and anterior cruciate ligament-return to sport after injury (ACL-RSI).

**Results:**

A total of 439 patients were included in the study, of whom 383 (87.2%) were male and 56 (22.8%) were female. The mean age at surgery was 28.06 ± 8.93 years. The mean time from injury to surgery was 97.68 ± 127.81 weeks, while the mean follow-up was 42.83 ± 39.22 months. Of 312 patients who completed the follow-up and reported to be sportsmen before surgery, 184 (58.97%) returned to the same or higher pre-injury level, 58 (18.58%) returned to a lower level, while 69 (22.11%) did not return to sports activity. The author analysed the Tegner score in three studies and noted a decrease compared to the pre-injury level (from 7.12 ± 0.8 pre-injury to 4.59 ± 0.57 at the final follow-up; p < 0.001). At the final follow-up, 4 studies analysed IKDC with a mean value of 75.14 ± 9.6, 3 reported a mean Lysholm of 51.81 ± 27.6, and two reported a mean ACL-RSI of 64.82 ± 0.149. Among the 439 patients, a total of 90 (20.5%) complications/re-operations were reported, while a total of 29 (6.6%) failures were recorded.

**Conclusions:**

Return to sport after MLKI occurs in approximately 75% of surgically treated patients, though return to high-level sport is about 60% of the patients. Furthermore, one in five patients report complications, while the failure rate is relatively low (< 7%).

**Level of Evidence:**

Systematic review of level 4.

## Introduction

Multiligament knee injuries (MLKIs) are characterised by the simultaneous rupture of two or more of the primary knee ligaments, namely the anterior cruciate ligament (ACL), posterior cruciate ligament (PCL), medial collateral ligament (MCL), and lateral collateral ligament or fibular collateral ligament (FCL). These severe injuries are not very common, with more recent reports indicating a rate of 0.072 occurrences per 100 patient-years in the general population. MLKIs, or multiligament knee injuries, are commonly linked to knee dislocation [1]. Knee dislocation is characterised by the complete tearing of both cruciate ligaments, with or without severe grade III damage to one of the collateral ligaments. The disruption of the popliteal artery and/or common peroneal nerve (CPN) is a critical medical situation that can lead to severe consequences if not identified promptly [2]. Younger patients are more frequently observed to have 6 MLKIs, with an average age of 37 years. There is a negative correlation between age and the occurrence of knee dislocation. MLKIs, or Musculoskeletal Lower Limb Injuries, are frequently linked to severe trauma, such as motor vehicle accidents or falls from a significant height [3]. However, around 50% of these injuries are caused by low-velocity mechanisms, with sports being the most common cause. The incidence rates of MLKI resulting from skiing activities and ball sports are 29.4% and 6.9%, respectively [4]. Motor vehicle accidents comprised only 19.2% of all injuries within the same group. Due to the infrequency of MLKI and the absence of agreement on the optimal clinical approach for this injury, the available MLKI clinical outcome studies are mostly confined to collections of cases with different treatment procedures. Moreover, the reporting of results after treating MLKI lacks standardisation. Some studies rely on patient comments regarding their ability to resume activities like employment, sports, and walking, while others focus on complication rates or subjective levels of patient satisfaction [5].

The majority of MLKI treatment outcome studies had a restricted number of participants, resulting in a limited ability to draw firm conclusions about future outcomes. According to one comprehensive review of MLKI, it has been found that cruciate ligament repair may have the same effectiveness as reconstruction in terms of symptoms reported by patients. Additionally, two other comprehensive analyses indicate that surgical treatment of MLKI leads to better rates of returning to work and participating in sports. Nevertheless, the impact of additional factors on the rates of returning to sports, apart from the choice between surgery and nonoperative treatment, is still uncertain [6]. The objective of this study was to conduct a comprehensive assessment of MLKI outcome studies to ascertain the overall rates of return to sport following MLKI.

## Material and Methods

The current systematic review was performed following the Preferred Reporting Items for Systematic Reviews and Meta-Analyses (PRISMA) guidelines [7].

### Eligibility Criteria

The literature selected for this study was based on the following criteria.

*Study Design*: Randomised controlled trials (RCTs), controlled (nonrandomised) clinical trials (CCTs), prospective and retrospective comparative cohort studies, case‒control studies and case series were included. Case reports and case series that did not report data on return to sports were excluded.

*Participants:* Studies conducted on skeletally mature patients treated surgically for MLKI.

*Interventions: *Studies that reported data on return to sports activity and level of return in patients treated surgically for MLKI. For MLKI treatment, the surgical technique and rehabilitation protocol were collected.

*Types of Outcome Measures:* The outcome measures extracted from the studies were rate of return to sport, level of return, complications, revision surgery Lysholm, Tegner, anterior cruciate ligament-return to sport after injury (ACL-RSI). and IKDC.

*Information Sources and Search:* A systematic search for relevant literature was performed in the PubMed (MEDLINE), Scopus, EMBASE and Cochrane Library databases of all studies published in English from January 1990 to May 2024. Two independent reviewers (RD and SBSK) assisted in conducting and validating the search. The following search terms were entered into the title, abstract, and keyword fields: “multiligament knee” or “MLKI" AND “return to sport” or “sports activity” “athletes” or “sports” or “sportsman”. Finally, only papers published in English were included.

### Data Collection and Analysis

*Study selection*: The retrieved articles were first screened by title and, if found relevant, screened further by reading the abstract. After excluding studies that did not meet the eligibility criteria, the entire content of the remaining articles was evaluated for eligibility. To minimise the risk of bias, the authors reviewed and discussed all the selected articles, references, and articles excluded from the study. In case of any disagreement between the reviewers, the senior investigator made the final decision. At the end of the process, further studies that might have been missed were manually searched by going through the reference lists of the included studies and relevant systematic reviews.

*Data collection process:* The data were extracted from the selected articles by the first two authors using a computerised tool created with Microsoft Access (Version 2010, Microsoft Corp, Redmond, Washington). Each article was validated again by the first author before analysis. For each study, data regarding the patients were extracted (age, sex, sports practised), their injuries (type, aetiology), the surgical technique, rehabilitation protocol, return to sport, level of postoperative activity, rate of complications, new surgeries, return to sport after revision surgery and clinical outcomes.

*Level of Evidence:* The Oxford Levels of Evidence set by the Oxford Centre for Evidence-Based Medicine were used to categorise the level of evidence [8].

*Evaluation of the Quality of Studies:* The quality of the selected studies was evaluated using the Methodological Index for Nonrandomized Studies (MINORS) score. The checklist includes 12 items, of which the last four are specific to comparative studies. Each item was given a score of 0–2 points. The ideal score was set at 16 points for noncomparative studies and 24 for comparative studies [9].

## Results

**Fig. 1:**
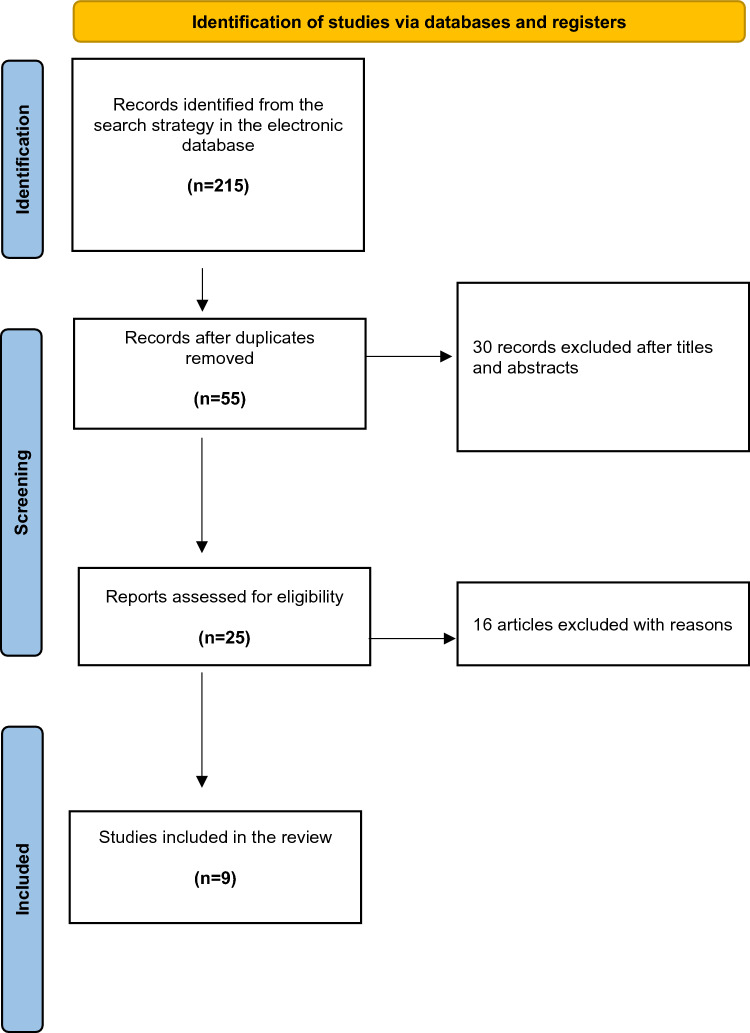
PRISMA (Preferred Reporting Items for Systematic Reviews and Meta-Analyses) flow chart indicating research article inclusion for final analysis

The electronic search yielded 215 studies. The 160 duplicates were removed, leaving 55 studies, out of which 30 were excluded after reviewing the abstracts, with a final number of 25. An additional 16 articles were excluded based on the aforementioned inclusion and exclusion criteria. This left nine studies for analysis [10–18]. Figure 1 shows the flowchart depicting the selection process for the studies. The analysed studies had a mean MINORS score of 10.6 (range 10–12), confirming the available literature's methodological quality (Table 1).

**Table 1:** Characteristics of the selected studies

Lead author	LOE	Minors score	Patients M/F	Age, y, Mean ± SD (range)	Time between injury and surgery	Follow-up, Mean ± SD (range)	Ligaments involved	Type of injury
Blokland et al. 2021 [10]	IV	10	31–18/13	30.9 ± 12.2	4 ± 4 months	Minimum 1 year	14 PCL + PLC 4 PCL + ACL 4 PCL + ACL + MCL 3 PCL + ACL + MCL + PLC 2 PCL + ACL + PLC 2 PCL + MCL 2 ACL + MCL + PLC	17 Sports 8 Traffic 1 Wowk 5 Other
Li et al. 2023 [11]	IV	10	33–21/12	48.6 ± 6.7	42 ± 71 weeks	59.1 (24–133) months	12 ACL + MCL 4 ACL + PLC 3 ACL + MCL + PLC 2 PCL + MCL 1 ACL + PCL 2 ACL + PCL + MCL 5 ACL + PCL + PLC 1 ACL + PCL + PLC + MCL	22 High 10 Low 1 Ultra-low
Bakshi et al. 2018 [12]	IV	10	50–50/0	n.a	n.a	388.71 ± 198.52 days	23 ACL + MCL 27 ACL + PCL/LCL	50 Football
Hecker et al. 2022 [13]	IV	11	22–17/5	45 ± 15	11 ± 8 weeks	49 ± 16 months	1 Schenk 2 9 Schenk 3-MCL 5 Schenk 3 – LCL 4 Schenk 4	8 Polytrauma 14 Monotrauma
Fine et al. 2023 [14]	IV	10	30–24/6	18.07 ± 2.532	94.7 ± 61.8 months	7.8 years (1.1 – 19.5)	10 ACL + MCL 7 ACL + PLC 3 ACL + LCL 5 ACL + MCL + PCL 1 PCL + PLC 1 ACL + PCL + LCL 1 ACL + LCL + PLC 1 ACL + MCL + LCL 1 MCL + PCL	Sports activity
Pizza et al. 2023 [15]	IV	10	42–39/3	31.4 ± 12.2	3.3 ± 7.3 years	9.6 ± 5.6 years	n.a	n.a
Cain et al. 2024 [16]	IV	11	53–53/0	18.1 ± 2.7	13.0 ± 12.7 days	7.7 ± 4.0 years	39 Two ligaments 13 Three ligaments 1 Four ligaments	n.a
Lee et al. 2020 [17]	IV	11	42–32/10	28.9 ± 10 y	20 acute 22 delayed	Minimum 1 year	16 KD 1 1 KD 2 15 KD 3 7 KD 4 3 KD 5	25 High energy 17 Low energy
Borque et al. 2021 [18]	IV	12	136–129/7	24.5 ± 4.4	n.a	12.8 ± 5.2 months	50 ACL + LCL 48 ACL + MCL 3 ACL + LCL + MCL 11 PCL + LCL 5 PCL + MCL 18 ACL + PCL 1 LCL + MCL	65 Football 59 Rugby 4 judoka 3 Gymnasts 2 Acrobats 1 Basketball 1 cricket 1 netball

*ACL* anterior cruciate ligament, *LCL* lateral collateral ligament, *MCL* medial collateral ligament, *PCL*  posterior cruciate ligament, *PLC* posterolateral corner, *KD*  knee dislocation

### Demographic Data

A total of 439 patients were included in the study, of which 383 (87.2%) were male and 56 (22.8%) were female. The mean age at surgery was 28.06 ± 8.93 years. The mean time from injury to surgery was 97.68 ± 127.81 weeks, while the mean follow-up was 42.83 ± 39.22 months. Details regarding the type of injuries and ligaments involved are reported in Table 1. Details regarding the surgical procedure and rehabilitation protocol have been reported in Table 2.

**Table 2:** Surgical and rehabilitation protocol

	Surgical technique	Rehabilitation protocol
Blokland et al. 2021 [10]	One-stage ligament reconstruction. MCL was treated with an internal brace in the acute setting, sometimes combined with an auto- or allograft augmentation or reconstruction. Autografts are used as much as possible, but allografts are often also needed for multiligament reconstructions	n.a
Li et al. 2023 [11]	n.a	n.a
Bakshi et al. 2018 [12]	n.a	n.a
Hecker et al. 2022	Primary repair of the torn ligaments and support with suture augmentation	Knee brace, flexion limits were 30, 60, and 90 for 2 weeks each. During the first 6 weeks, partial weightbearing of 15 kg was allowed. Subsequently, a stepwise load increase to full weightbearing was initiated over a further 6 weeks
Fine et al. 2023 [14]	n.a	n.a
Pizza et al. 2023 [15]	n.a	n.a
Cain et al. 2024 [16]	ACL: BTPB PCL: repair with or without augmentation MCL/PMC: primary repair LCL/PLC: repair	Drop lock brace set at 0° of extension or 10° of flexion (if the PLC and LCL were injured). All patients were permitted to be < 50% weightbearing using 2 crutches, with range of motion permitted between 0° and 45°. At approximately 12 days postoperatively, patients progressed to 75% weightbearing status. In the case of PLC/LCL injury, full extension was allowed at weeks 3 to 4. Between weeks 6 and 8 (depending on severity of injury), patients progressed to full weightbearing and were provided a functional ACL/PCL brace
Lee et al. 2020 [17]	Allografts were used in all cases of PCL, PLC, and PMC reconstruc- tion. For the anterior cruciate ligament (ACL), 24 ACL reconstructions used quadriceps autograft, and 10 cases were reconstructed with quadriceps allograft on the basis of patient ages and activity levels. The Compass Knee Hinged External Fixator (Smith & Nephew, Memphis, TN) was used in high-grade injury (total 12 patients, 28.5%), six KDIII and six KDIV, to provide increased stability and protect recon- structed grafts during initial healing	Patients begin ROM at 0 to 30 degrees and progress as tolerated. At 3 to 4 weeks, the hinged knee brace is unlocked during weight- bearing activities. Physical therapy starts after the first 2 weeks. The main focus during the initial recovery period is to obtain and maintain motion. By 6 weeks, the patient should be expected to have 0 to 90 degrees of active and passive knee motion, good patellar mobility, and normal gait without crutches
Borque et al. 2021 [18]	ACL: anteromedial portal with single bundle BPTB or six strand hamstring PCL: six-strand transtibial hamstring Collateral ligament: repair and/or reconstruction	Weight bearing for 2 weeks followed by 2 weeks of partial weight bearing. ACL-based injuries were placed in a hinged knee brace for 6 weeks with no restrictions on flexion. PCL-based and bicruciate injuries were placed in a hinged knee brace locked in full extension in the operating room and were then transitioned to a dynamic PCL brace when tolerated, usually 2–3 days post-operatively. The dynamic PCL brace was worn for 3 months with flexion limited to 90° for the first 4 weeks, then slowly increased to full flexion at 12 weeks

*ACL *anterior cruciate ligament, *PCL* posterior cruciate ligament, *PCL* posterolateral corner, *MCL* medial collateral ligament, *PMC* posteromedial corner, *LCL* lateral collateral ligament, *PLC* posterolateral corner, *KD* knee dislocation

### Return to Sport

Of 312 patients who completed the follow-up and reported to be sportsmen before surgery, 184 (58.97%) returned to the same or higher pre-injury level, 58 (18.58%) returned to a lower level, while 69 (22.11%) did not return to sports activity. The authors analysed the Tegner score in three studies and noted a decrease compared to the pre-injury level (from 7.12 ± 0.8 pre-injury to 4.59 ± 0.57 at the final follow-up; p < 0.001). At the final follow-up, four studies analysed IKDC with a mean value of 75.14 ± 9.6, three reported a mean Lysholm of 51.81 ± 27.6, and two reported a mean ACL-RSI of 64.82 ± 0.149.

### Complications

Among the 439 patients, 90 (20.5%) complications/re-operations were reported, of which 7 (1.59%) infections, 15 (3.41%) hardware removal, 41 (9.33%) stiffness, 1 (0.22%) meniscal allograft transplantation, 11 (2.5%) meniscal or chondral treatment, 14 (3.18%) cyclops syndrome, and 1 (0.22%) osteochondral allograft. Details are reported in Table 3.

**Table 3:** Return to sport, complications and failures

	Return to sport		Failures	Complications and reoperations
Blokland et al. 2021 [10]	6 Returned to their pre-injury level 17 Returned to lower level 2 No return to sports 1 Unknown		1 ACL revision 1 Total knee arhtroplasty	1 Arthroscopy for infection 2 Hardware removal
Li et al. 2023 [11]	IKDC 59.5 Lysholm 69.7 Tegner pre: 6.1 / Tegner post: 3.8 Patients practiced sports pre injury: 17 After injury: - 1 same level - 7 lower level		2 Total knee arhtroplasty	5 Stiffness (4 underwent manipulation under anesthesia)
Bakshi et al. 2018 [12]	32 Returned to sports 15 Returned to their pre-injury level		n.a	n.a
Hecker et al. 2022 [13]	Preinjury: Tegner 7	Final follow-up: Tegner 5 Lysholm 84 IKDC 73 ACL-RSI 65	2 ACL revisions 1 PCL revisions 2 MCL revisions 2 LCL revisions	7 Arthroscopic arthrolysis for stiffness 1 Meniscal allograft transplantation 1 Arthroscopy for infection
Fine et al. 2023 [14]	27 returned to sports 13 returned to pre-injury or higher level ACL-RSI: 64.7 IKDC-SF: 78.3 SF-12 physical: 51.2		n.a	n.a
Pizza et al. 2023 [15]	Pre-injuyr: Tegner 8 Lysholm: 43.5	Final follow-up: Tegner 5 Lysholm 20.9	6 Surgical failure	n.a
Cain et al. 2024 [16]	IKDC 84 Return to sport 32 Yes 21 No		4 Ligament revisions	1 Arthroscopy for arthrofibrosis 3 Arthroscopy for meniscal/chondral treatment 1 Hardware removal
Lee et al. 2020 [17]	Significant improving in physcal function		3 PLC revisions 1 total knee arthroplasty	3 Arthroscopy for infection 3 Hardware removal 3 Arthroscopy for arthrofibrosis
Borque et al. 2021 [18]	117 returned to higher level 3 Returned to lower level		4 Revision ligament	25 Manipulation for arthrofibrosis 14 Debridement for cyclops 9 Hardware removal 7 Partial meniscectomies 1 Revision lateral meniscal repair 1 Osteochondral allograft 2 Arthroscopy for infection

*IKDC* international knee documentation committee, *ACL-RSI* anterior cruciate ligament return to sport after injury, *SF* short form, *ACL* anterior cruciate ligament, *PLC* posterolateral corner, *PCL* posterior cruciate ligament, *MCL* medial collateral ligament, *LCL* lateral collateral ligament

### Failures

A total of 29 (6.6%) failures were reported, of which 25 (5.69%) were ligament failures, and four (0.91%) were converted to total knee arthroplasty. Details are reported in Table 3.

## Discussion

The most important findings of the current study reveal that more than 75% of the patients return to practice sports after MLKI, but only about 60% return to their pre-injury level. Furthermore, one in five patients report complications, while the failure rate is relatively low (< 7%). Literature on MLKI is still scarce, with few quality studies considering the low incidence of this kind of injury. In 2018, Everhart et al. systematically reviewed MLKI outcome studies to determine overall rates of return to work or sport after MLKI and risk factors for lack of return to work or sport after MLKI [6]. A total of 524 patients (21 studies) were included. Return to high-level sport was low (22%-33%). Return to any level of sport was 53.6% overall (178/332), with a higher rate reported in studies with all surgical patients (59.1%, 114/193 patients) versus studies with mixed surgical and nonoperative treatment (46.0%, 64/139 patients). The rate of return to work with little or no modifications was 62.1%, and the return to any work was 88.4%. Obese patients had lower postoperative Tegner scores than the general population. Among studies without Schenck grade IV and V injuries, return to work with no or minimal modifications (100%) was higher than studies including grade IV and V patients (66.0%). Return to any work was higher in studies without vascular injuries (96.3%) versus those including them (80.2%) [6].

Multiligament knee injuries can occur due to high-, low-, and ultra-low-velocity traumas. High-velocity injuries typically result from motor vehicle accidents, falls from great heights, or severe crush injuries. These types of injuries are more prone to being accompanied by additional injuries. Most knee dislocations with modest velocity occur during sports or falls from heights less than 1.5 m. These cases generally have more favourable results. Ultra-low velocity multiligament knee injuries primarily affect individuals who are obese and are frequently incurred during routine everyday activities. Due to the ongoing obesity epidemic, there has been an increase in the occurrence of these injuries. This has resulted in a significant rise in the number of fat older individuals, as well as young patients with injuries caused by high levels of force. Examining obese patients might be particularly difficult because of their increased incidence of related injuries and postoperative problems compared to non-obese patients [1–3].

MLKI in morbidly obese patients appears to be a separate clinical condition with much poorer functional outcomes compared to the general MLKI group. Female patients are more prone to obesity-related injuries, which typically arise from low-velocity incidents. The unfavourable functional outcomes described in this analysis are corroborated by the low satisfaction rates documented by Werner et al. for severely obese patients who received treatment for MLKI resulting from a low-energy mechanism. After an average of 5.8 years, 12 out of 17 patients reported being dissatisfied or highly dissatisfied. This could be partially attributed to the delayed acknowledgement of MLKI and an increased probability of nonoperative intervention. While grossly obese patients with MLKI who got surgical treatment experienced improved range of motion and reduced instability compared to those who received non-surgical treatments, the occurrence of perioperative complications was much higher in the morbidly obese population [19].

Our systematic review found different rehabilitation approaches that can lead to different timings for returning to sport. Objective measures such as strength, balance, and power and laboratory-based assessments of functional movement patterns (such as squatting and hopping) are infrequently documented in studies involving this particular group [20]. These metrics frequently serve as the primary criteria for determining whether an individual can return to sports, particularly in cases of a common knee injury such as an anterior cruciate ligament rupture. The limited number of research examining functional movement patterns after multiligament knee injuries primarily concentrates on walking. Only one study provides information on the strength of the quadriceps muscle [21]. These criteria are crucial for promoting healthy human movement and athletic function. However, they are not clearly defined in the current literature on multiligament knee injuries. This lack of clarity makes it challenging to understand and interpret reported results, such as the development of knee osteoarthritis or the rate at which individuals return to sports activities. Considering the reported connections between the advancement of osteoarthritis and the weakening of the quadriceps muscles, we cannot attribute multiligament knee reconstruction as the sole cause of osteoarthritis. Instead, it is more likely to be one of several contributing factors, including ongoing muscle weakness and incorrect movement patterns [22].

The literature lacks sufficient rehabilitation guidelines specifically tailored to multiligament knee injuries, emphasising the importance of early and effective rehabilitation followed by gradual advancements in exercise programme design and intensity for successful return to sport. Furthermore, there is a lack of established rules and objective criteria for determining whether individuals in this community can safely return to participating in sports [23].

Most patients can resume their work duties following an MLKI, although the recovery period may extend over several months or even longer. Adjustments to their work environment or tasks may be necessary in other cases. Ibrahim et al. recommended that they resume sedentary work within 8 to 10 weeks and a more physically demanding job within 7 to 10 months. Mariani et al. also found that patients refrained from engaging in strenuous physical work for around six months. This could be attributed to the fact that the patients who get MLKI are frequently young and may have physically demanding jobs. Outcomes can differ When counselling older patients or patients with less physically active jobs [24].

## Limitations

A limitation of this systematic review is that it relies on data from studies with Level III and IV evidence. This is because there is a scarcity of high-level evidence research specifically connected to multiligament knee injuries. The research analysed in this review focused on medical centres that handle a large number of MLKIs. It is possible that untrained physicians may have less favourable results while treating this complicated injury than reported in the current analysis. Due to the unavailability of patient-level data, we could not adequately account for the impact of injury severity, obesity, nonoperative treatment, vascular injury, or peroneal nerve injury on the resumption of sports or work. The utilisation of summary data from diverse patient populations hinders the thorough categorisation of these risk variables and introduces a one-sided bias. In particular, this evaluation is likely underestimating the adverse impact of these characteristics on the ability to resume work or engage in sports.

Furthermore, due to constraints in the quality of reporting, we could not compare outcomes based on the status of cartilage injury. The current evaluation did not fully recognise the potential negative impact of peroneal nerve injury, especially complete injury, on the ability to return to work or engage in sports. Due to numerous discrepancies in treatment protocols, we could not evaluate the impact of parameters such as rehabilitation procedure or graft selection on outcomes.

## Conclusions

Return to sport after MLKI occurs in approximately 75% of surgically treated patients, though return to high-level sport is about 60% of the patients. Furthermore, one in five patients report complications, while the failure rate is relatively low (< 7%).

## Data Availability

The raw data are available upon reasonable request to the corresponding author.

## Code Availability

The datasets generated during and/or analysed during the current study are available from the corresponding author on reasonable request.
